# Anti-inflammatory therapy of atherosclerosis: focusing on IKKβ

**DOI:** 10.1186/s12950-023-00330-5

**Published:** 2023-02-23

**Authors:** Jiali Gan, Lin Guo, Xiaolu Zhang, Qun Yu, Qiuyue Yang, Yilin Zhang, Wenyun Zeng, Xijuan Jiang, Maojuan Guo

**Affiliations:** 1 School of Integrative Medicine, Tianjin University of Traditional Chinese Medicine, Tianjin, China; 2 Oncology department, Ganzhou People’s Hospital, Ganzhou, Jiangxi China

**Keywords:** Atherosclerosis, IKKβ/NF-κB, Natural product-based derivatives, Natural extracts, Synthetic drugs

## Abstract

Chronic low-grade inflammation has been identified as a major contributor in the development of atherosclerosis. Nuclear Factor-κappa B (NF-κB) is a critical transcription factors family of the inflammatory pathway. As a major catalytic subunit of the IKK complex, IκB kinase β (IKKβ) drives canonical activation of NF-κB and is implicated in the link between inflammation and atherosclerosis, making it a promising therapeutic target. Various natural product derivatives, extracts, and synthetic, show anti-atherogenic potential by inhibiting IKKβ-mediated inflammation. This review focuses on the latest knowledge and current research landscape surrounding anti-atherosclerotic drugs that inhibit IKKβ. There will be more opportunities to fully understand the complex functions of IKKβ in atherogenesis and develop new effective therapies in the future.

## Introduction

Arteries are the conduits that transport blood from the heart to tissues and organs [1]. The artery wall, such as aortic wall, is made up of three layers from inside to outside: the intima, media, and adventitia. The intima consists mainly of a single layer of endothelial cells (ECs) and a thin basal membrane that acts as a barrier to prevent the leakage of blood components into the vessel wall. The media layer regulates the artery elasticity, which is primarily composed of smooth muscle cells (SMCs). The adventitia refers to the connective tissue covering the outer layer [2]. Increased thickness of the vessel wall, stenosis, or occlusion of the lumen may lead to ischemia and dysfunction of tissues and organs. Atherosclerosis is a common cause of ischemic diseases (such as stroke and myocardial ischemia) with high morbidity and mortality worldwide [3]. It is characterized by the formation of atherosclerotic plaques in the intima of large or medium-sized systemic arteries.

It is well established that atherosclerosis is not only a metabolic disorder, but also a chronic low-grade sterile inflammation in the vasculature orchestrated by a network of inflammatory cytokines. ECs, macrophages and migratory SMCs from the media layer are the major cellular components of atherosclerotic lesions [4–6]. These cells cooperate to initiate the inflammatory signal and to upregulate the adhesion molecule expression and athermanous plaque is finally formed [7]. Recently, it has demonstrated that nuclear factor-κappa B (NF-κB) is closely related to atherosclerosis-associated inflammation [8, 9]. The researchers found that NF-κB was activated in the key components of atherosclerotic plaques, including ECs, macrophages, and SMCs [10–12]. IκB kinase β (IKKβ), the predominant catalytic subunit of the IKK complex [13], is required for the canonical activation of NF-κB, which also known as a critical molecular link between inflammation and atherosclerosis [14]. Fortunately, a variety of natural product-based derivatives, natural extracts, synthetic drugs, as well as peptides et.al other drugs, all display anti-atherogenic potential by inhibiting IKKβ-mediated inflammation, which may be potential therapy medicine for atherosclerosis. As a result, this review provides the current findings on IKK and atherosclerosis, as well as outlines therapeutic interventions used to target IKK for the treatment of atherosclerosis.

## Inflammation and atherosclerosis

**Fig. 1:**
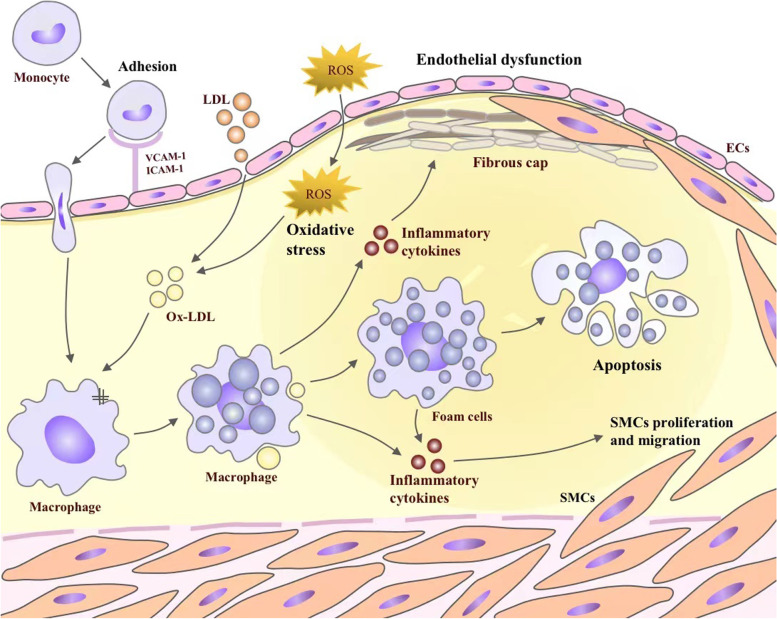
The key inflammatory mechanisms involved in the development of atherosclerosis. Monocytes are first recruited to developing plaques by VCAM-1, ICAM-1, and E-seletin. Then, monocytes differentiate into macrophages, and reactive oxygen species (ROS) from vascular lumen accumulates oxidizes LDL (ox-LDL). Ox-LDL is mostly taken up by macrophage scavenger receptors and becomes foam cells. Macrophages and foam cells secrete inflammatory cytokines, such as IL-6, IL-12 and TNF-α, which in return exacerbates the inflammatory response. Inflammatory cytokines secrete matrix metalloproteinases (MMPs), which degrades the fibrous plaque. This could lead to plaque rupture and thrombosis. In addition, inflammatory cytokines promote the proliferation and migration of smooth muscle cells (SMCs), which contributes to the formation of fibrous cap. Foam cell apoptosis promotes plague rupture

Atherosclerosis, a chronic inflammatory disease of the vessel wall, is characterized by the accumulation of lipid-laden macrophages and fibrous material in the large or medium-sized systemic arteries [15]. A key initiating event is the retention of ApoB-containing lipoprotein particles under the endothelial layer of the arterial wall [16]. There is overwhelming evidence that a transgenic expression of a natural antibody to oxidized phospholipids suppresses lesions in hypercholesterolemic low-density lipoprotein receptor knock out (LDLR^*−/−*^) mice, which supports the lipid oxidation hypothesis for atherosclerosis [17]. What's more, as a result of the disturbances in blood flow, the ECs are activated, and the tight junctions between them become "leaky", which facilitates the trans-endothelial transport of plasma LDL and TG-rich lipoproteins or diffusion at cell–cell contact points, and reaches the intima [18]. As a matter of fact, the subsequent activation of ECs is caused by the oxidation of lipoprotein lipids and other mediators of inflammation, which leads to the expression of vascular cell adhesion molecule 1 (VCAM-1), intercellular cell adhesion molecule-1 (ICAM-1), and E-selectin, as well as promotes the adhesion of monocytes and chemokines, and inflammation occurs [19]. This process involves many cells and cytokines, such as ECs, macrophages, lymphocytes (T and B cells), dendritic cells (DCs), interleukin family, adhesion molecules, and TNF-α [20]. Immediately afterwards, monocytes are recruited to the vessel wall and enter the intimal, which are stimulated by a macrophage-stimulating factor (M-CSF) and other cytokines to differentiate into macrophages [21]. In response to local microenvironmental signals, macrophages acquire functionally distinct phenotypes, including the pro-inflammatory M1-like phenotype, and the pro-resolving M2-like phenotype [22]. Macrophages contribute very importantly to lesion progression, in ApoE^−/−^ mice, the number of macrophages in early atherosclerosis is determined by recruitment; in more advanced lesions, however, it was largely dependent on proliferation of local macrophages, rather than monocyte [23, 24]. At the same time, in response to lipid oxidation, LDL transforms into ox-LDL, which is scavenged by monocyte receptors upon infiltration, converting monocytes into lipid-filled macrophage foam cells [25]. With the lesion progresses, SMCs in the media transform from a contractile to a proliferative state, and migrate into the intima [26]. Eventually, the intimal SMCs secrete an extracellular matrix mainly composed of collagen, forming a fibrous cap to protect against plaque rupture. A study of lineage tracing shows that the intimal SMCs can differentiate into macrophage-like and osteochondrogenic descendants [27]. In the presence of lipid, macrophage-like SMCs can produce foam cells, accumulated foam cells undergo apoptosis and inhibited efferocytosis [28]. It is inevitable that some apoptotic foam cells may escape efferocytosis and contribute to the formation of necrotic lipid cores, causing secondary necrosis and inflammation [29] (Fig. 1).

## The role of IKKβ/NF‑κB in the development of atherosclerosis

**Fig. 2:**
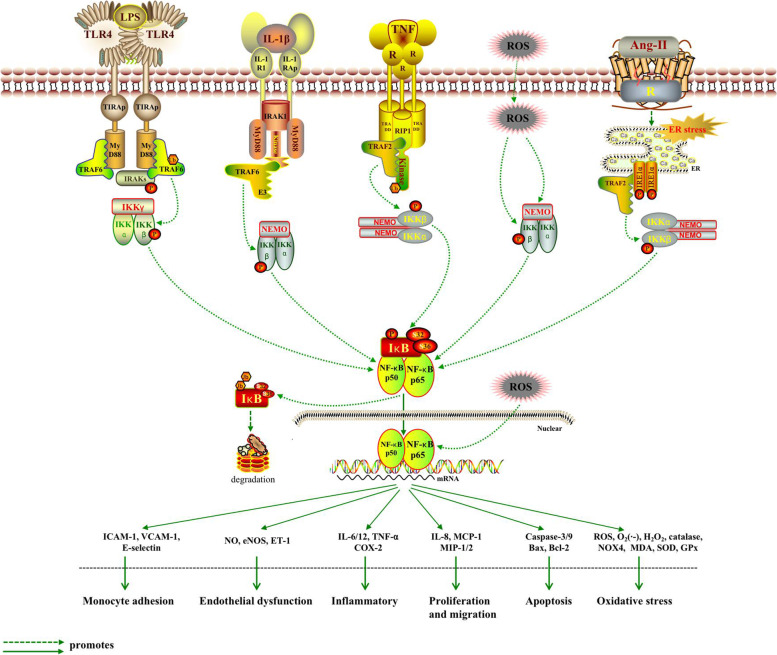
The Activation and regulation of IKKβ/NF-κB pathway. ①The binding of LPS to TLR4 recruits TIRAP. Then MyD88 joins the comlex which is bound by IRAKs and TRAF6 to activate IKKβ. ② MyD88 is recruited upon binds of IL-1 to IL-1RI. Then, IRAK1 comjoins the complex and TRAF6 also assemble to IKK complex. ③ The bingding of TNF with TNFR leads to the binding of TRADD, TRAF2 with the protein kinase RIP1, which forms a platform for the recruitment of TRAF2. When ubiquitinated RIP1 bindings to NEMO, it phosphorylates and activates IKKβ. ④ ROS from vascular lumen interact with various elements of the IKK/NF-κB signaling pathway. On the other hand, the phosphorylation of p65 in which ROS are involved leads to a greater activation of NF-κB. ⑤ The binding of ang II induces endoplasmic reticulum (ER) stress, activates and phosphorylates inositol-requiring 1α (IRE1α). Phosphorylated-IRE1α (p-IRE1α) recruits TRAF2, and then activates IKKβ. In the canonical pathway, IκBα is phosphorylated in an IKKβ- and NEMO(IKKγ)-dependent manner, which results in the nuclear translocation of mostly p65 or p50-containing heterodimers. Then, IκB degradates. The transcriptional p65 and p50 stimulate the production of inflammatory cytokines and finally monocyte adhesion, endothelial dysfunction, inflammatory, SMCs proliferation and migration, apoptosis, and oxidative stress all ensue. TIRAP: Toll/IL-1 receptor adaptor protein; MyD88: myeloid differentiation primary response gene 88; TRAF6: TNF-R-associated factor 6; IRAKs: IL-1 receptor-associated kinases; TRADD: TNF-R-associated death domain; RIP1: receptor-interacting protein 1; TRAF2: TNF-R-associated factor 2

The NF‑κB signaling pathway consists of NF‑κB, the inhibitor of Kinase B (IκB), the IκB kinase (IKK) complex and IKK upstream kinases [30]. There are two main pathways involved in NF-κB activation, namely the canonical (classic) and the non-canonical pathways [31]. The canonical NF-κB pathway is present in most cell types. The most abundant forms of NF-κB activated by the typical pathway are the heterodimers of p50 and p65 [32]. In the resting state, its binding to IκB keeps NF-κB in inactive form in the cytoplasm when nuclear translocalization signals [33, 34]. When cytokines, such as TNF-α, IL-1, and lipopolysaccharide (LPS), attach to their receptors, TNFR, IL-1R, and toll- like receptor (TLR), respectively, IKK is activated (Fig. 2) [35]. Then, IKK induces phosphorylation of IκB on Ser32 and (or) Ser36, and subsequent polyubiquitination. As a result, NF-κB dissociates from the NF-κB/IκB complex, and translocates to nucleus, where it stimulates the transcriptions of cytokines and cell adhesion molecules. IKK consists of two catalytic subunits, IKKα and IKKβ, and an NF-κB essential modifier (NEMO), also known as IKKγ [36]. There is an NEMO-binding domain (NBD) at the C-terminus of IKKα and IKKβ, which mediates the formation of the IKK complex. Although IKKα and IKKβ have similar structural features, they work in different ways. During activation of the canonical pathway, IKKβ is the dominant kinase promoting phosphorylation of IκB on Ser32 and Ser36, instead of IKKα [37]. Once the serine on the activation loop of IKKβ is mutated to alanine, TNF-α, IL-1, and LPS all fail to activate NF-κB [38]. In contrast, the same mutation in IKKα failed to reveal a similar effect [39]. In summary, the IKKβ/NF-κB pathway plays a pivotal role in pro-inflammatory responses, and therefore IKKβ inhibitors may be an effective target in modulating NF-κB activity.

During atherosclerosis, NF-κB activation is dependent on IKKβ rather than IKKα [40]. A small peptide mimicking NBD structure was synthesized and introduced into cells, which significantly inhibited NF-κB activity. In diabetic mice, NBD peptide attenuated NF-κB activation and markedly reduced the size of atherosclerotic plaques by inhibiting IKK complex formation [41]. Similarly, ebselen reduced atherosclerotic lesions in the aorta by inhibiting the phosphorylation of IKKβ, thus abandoned NF-κB activation in diabetic ApoE^−/−^ mice [42]. Furthermore, myeloid-specific IKKβ deficiency alleviated atherosclerosis in LDLR^−/−^ mice [40]. IKKα deficiency did not attenuate atherosclerosis, but only haematopoiesis in ApoE^−/−^ mice [43]. However, some studies treported different conclusion. For instance, IKKβ deficiency did not affect atherosclerotic lesion size, rather it promoted plaque vulnerability and lesional inflammation in obese LDLR^−/−^ mice [44].

Despite some conflicting views, the anti-inflammatory therapy targeting IKKβ is regarded as a effective way in ameliorating atherosclerosis. A variety of drugs, especially natural products-based derivatives, natural extracts, and synthetic drugs, have been shown to inhibit IKKβ, therefore could be candidate drugs to treat atherosclerosis.

## The role of IKKβ in key cell types and the influence on atherosclerosis

### Endothelial cells IKKβ and atherosclerosis

In atherosclerotic plaques, IKKβ/NF-κB signaling induced by ox-LDL is activated in ECs as well as in vulnerable plaques [45–47]. What’s more, the activation of endothelial IKKβ stimulates monocyte infiltration into the arterial intima, thereby exacerbating atherosclerosis [47]. Shear stress [48], TNF-α [49], IL-1β [50], LPS [51], high glucose [52], and insulin resistance [52] are all related to the activation and high expression of IKKs in ECs. ECs are continuously exposed to the shear caused by blood flow, which activates NF-κB that is mediated by integrin/Flk-1, a receptor for VEGF (vascular endothelial growth factor)/IKK pathway [48]. Tripartite motif-containing 14 (TRIM 14) is positively regulated by TNF-α, IL-1β, and LPS, which in turn active NF-κB to form a positive feedback, and drives endothelial activation via the interaction between TRIM14 and NEMO [53]. Notably, TRIM 14 promotes endothelial activation by activating NF-κB to involve in the development of human atherosclerosis [53]. High glucose-induced endothelial dysfunction is accompanied by increased expressions of inflammatory cytokines and adhesion molecules, and adhesion molecules. Endothelial-monocyte adhesion is mediated by the CIKS (connection to IKK and SAPK/JNK), an upstream regulator of NF-κB [54]. Additionally, silver nanoparticles (AgNPs), a potentially hazardous factor for early atherosclerosis, were found to induce HUVECs impairment and dysfunction by activating the IKK/NF-κB pathways [55]. When IKKβ is persistently activated by expressing the dominant interfering mutant, most NF-κB target genes are maximally induced in human microvascular endothelial cell line-1 [56]. The opposite result was observed with dominant negative IKKβ or blocking IKKα/β in response to low shear stress in ECs [57].

### Macrophages IKKβ and atherosclerosis

Macrophages are known to play a major role in the development of atherosclerosis, which are not only the major pro-inflammatory cells, but also the essential cellular components of atherosclerotic plaques [58]. Phagocytic macrophages engulf large amounts of ox-LDL and transform into foam cells, which is a hallmark of atherosclerosis [59]. In the plaque microenvironment, there is a vicious cycle between macrophage infiltration and pro-inflammatory factor release [60]. Ox-LDL-activated macrophages upregulated the expression of IKKα and IKKβ, and similar results were found in macrophages induced by LPS in vitro [61, 62]. Clinical studies have shown that obstructive sleep apnea, characterized by intermittent hypoxia, is an independent risk factor for atherosclerosis, especially premature atherosclerosis. It is worth mentioning that its mechanism is closely related to the activation of IKKβ-dependent NF-κB pathway in murine macrophages [63, 64]. Further, excessive nutrition input activated the IKK/NF-κB signaling pathway and inflammation in macrophages, which was strongly attenuated by major vault protein (MVP), an upstream inhibitor of IKK [65].

Vulnerable atherosclerotic plaques are prone to become culprit plaques that cause acute coronary syndromes (ACS), such as acute myocardial infarction, a serious complication of atherosclerosis [66]. Histone deacetylase 9 (Hdac9), a member of the histone deacetylase II family, catalyzes the deacetylation of histone H3K16ac and other non-histone proteins, contributing to atherosclerosis and inflammation [67]. By activating IKK, Hdac9 increases lesional macrophage content and promotes vulnerable plaque formation, whereas hematopoietic Hdac9 knockout promotes the opposite role outcome [68]. Park SH, et al. generated myeloid-specific IKKβ-deficient LDLR^−/−^ mice and found that the lack of IKKβ in macrophage attenuated high-fat diet-induced atherosclerosis in LDLR^−/−^ mice mainly by alleviating inflammatory responses of macrophages [40]. Moreover, chronic uremia promoted atherosclerosis in uremic apoE^−/−^ mice by promoting endoplasmic reticulum (ER) stress-related inflammation, including activating ER stress induced inflammation via activating IKK phosphorylation [69]. Phosphorylated inositol-requiring 1α (p-IRE1α) is an ER stress marker protein expressed mainly in macrophages from atherosclerotic lesions. IRE1α‑siRNA inhibited inflammation and IKK phosphorylation in Ang II-treated RAW264.7 macrophages, thereby suppressing IκB degradation and NF-κB p65 nuclear translocation [69]. Activation of renin-angiotensin system (RAAS) also aggravated atherosclerosis in experimental renal failure apoE^−/−^ mice and upregulated IKK phosphorylation in Ang II-stimulated RAW264.7 macrophages. It suggested that the IKK/NF-κB pathway promotes ER stress-induced inflammation and atherosclerosis [70].

### Vascular smooth muscle cells IKKβ and atherosclerosis

During atherogenesis, VSMCs undergo a phenotypic transformation from contractile to synthetic upon the induction of reprogramming transcription factors, such as Krüppel-like factor4 (KLF4) and Octamer-binding transcription factor (OCT4) [27]. Synthetic VSMCs acquire the capacity to proliferate and migrate from the media into intima at the sites of plaques [71]. What’s more, VSMCs synthesize most of the interstitial collagens that stabilize the fibrous caps of plaques [72]. Unlike macrophages, VSMCs transform into a pro-inflammatory phenotype similar to macrophages, acting as both targets and sources of inflammatory factors [26, 73].

Similarly, an activated IKKβ-NF-κB axis has been observed in VSMCs from human atherosclerotic lesions [74]. In vitro, IL-1β-induced proliferation of VSMCs in human saphenous veins via IKKβ activation, which was attenuated by transfection of inactive IKKβ mutants [75]. Oxygen free radicals play a key role in atherogenesis by activating NF-κB in VSMCs, which associated with IKKβ-induced degradation of IκB [76]. Similar results were obtained in VSMCs stimulated by LPS or IL-1β in vitro [77]. IKKβ knockout in VSMCs by the SM22Cre-IKKβ-flox system lead to significant inhibition of vascular inflammation and atherosclerotic plaques in LDLR^−/−^ mice [14, 40]. Furthermore, IKKβ knockout in VSMCs induced by U0126 and SB202190 (inhibitors of p42/p44 MAPK) inhibited cytosolic phospholipase A2 (cPLA2) expression, which exacerbated the atherosclerosis-related vascular inflammation [78].

## Anti-inflammatory therapy targeting IKKβ in atherosclerosis

### Natural product-based derivatives

Vinpocetine, a derivative of the alkaloid vincamine, is one of the most commonly prescribed medicines for the treatment of cerebrovascular disease and cognitive impairment in many countries [79]. The results of a study revealed that vinpocetine inhibits atherosclerosis in ApoE^−/−^ mice by targeting the Akt/NF-κB receptor dependent pathway [80]. In addition, it has also been shown that vinpocetine is an IKK inhibitor, which inhibits IKK with an IC_50_ value of approximately 17.17 μM, thereby suppressing the NF-κB-dependent inflammation [81]. A growing body of evidence suggests that vinpocetine is anti-inflammatory in a variety of cell types by directly targeting of IKKβ, including ECs, VSMCs, and monocytes/macrophages [82].

Metformin is a biguanide developed from the guanidine derivative galegine found in *Galega officinalis* (*French lilac*), widely used for the treatment of type 2 diabetes mellitus [83]. According to preclinical and clinical studies, metformin has anti-inflammatory properties and performs a protective role in cardiovascular disease, including atherosclerosis [84, 85]. In the atherosclerosis model of rabbits, metformin impeded the atherosclerosis progression, which might be related to inhibiting the adhesion molecules and inflammatory factors by blocking the IKKβ/NF-κB translocation [86]. What's more, there is the conclusive evidence that metformin suppressed the TNF-α–induced phosphorylation of the upstream kinase site p176/17738 on IKKα/β [87]. A study reported that metformin pretreatment (100 ~ 1000 mmol/L) inhibited IKKα/β phosphorylation, IκB degradation, and ultimately IL-6 production in TNF-α-induced HUVECs via the PI3K-dependent AMPK phosphorylation [88].

Naringin is a plant-derived flavonoid, found inmany plants such as grape, citrus species, and fractus aurantii, which has potential for preventing atherosclerosis [89]. In ApoE^−/−^ mice fed a high-fat diet, naringin significantly alleviated atherosclerosis and reduced the serum and liver cholesterol levels by 24.04 and 28.37%, respectively [90]. Interestingly, in TNF-α-stimulated HUVECs, naringin suppressed the activation of NF-κB by inhibiting IKKβ activity [91, 92]. What's more, in a dose-dependent manner, naringin appears to reduce the risk of atherosclerosis by inhibiting the adhesion of THP-1 monocytes to TNF-α-stimulated HUVECs [91, 92].

Emodin is an anthraquinone derivative, naturally occurring in oriental herbs, with diverse biological properties [89]. It has been demonstrated by experimental studies that emodin is capable of attenuating and stabilizing atherosclerotic plaques [93]. Another study found that emodin exhibited inhibitory effects on LIGHT-induced macrophage migration, which was the result of NF-κB activation by NADPH oxidase p47 (phox), suggesting that its anti-atherosclerosis effect was attributed to interventing the IKK [94]. Additionally, emodin inhibited TNF-α-induced activation of NF-κB in rat aortic VSMCs and dose-dependently reduced inflammatory factor gene expression, supporting its anti-atherogenic effects [95].

Green tea polyphenols consist of more than 30 phenolic substances, the main components of which are catechins and their derivatives [96]. Extensive laboratory and epidemiological studies have demonstrated that green tea polyphenols reduce the risk of cardiovascular disease in both animals and humans [97, 98]. As a result of pretreatment with green tea polyphenols, oxLDL-induced proinflammatory cytokine TNF-α and NF-KB activation was reduced by inhibiting the IKK activity in a dose-dependent manner [99]. Stybenpropol A, a resin secreted from the styrax tonkinensis bark, has a protective effect on the vascular endothelium [100]. In vitro, stybenpropol A blocked the monocyte migration, as well as adhesion to TNF-α-induced HUVECs when it inhibited the IKK/NF-κB pathway [100]. Methyl-β-cyclodextrin (MβCD), a cyclodextrins derivative, due to its high affinity for cholesterol, it is one of the most effective agents for removing plasma membrane cholesterol [101]. By downregulating adhesion molecule expression via the LPS/IKK/NF-κB pathway, MβCD may be able to inhibit monocyte endothelial adhesion, which indicates MβCD may have anti-atherosclerosis effects [51].

### Natural extracts

Tanshinone IIA is a main lipophilic component derived from the root extract of *Salvia miltiorrhiza*, which has been widely used in traditional Chinese medicine for the treatment of cardiovascular diseases [102, 103]. According to a study, Tanshinone IIA downregulated the NF-κB activity, and reduced the expression of TNF-α and MCP-1, to stabilize vulnerable atherosclerosis plaque in ApoE^−/−^ mice [104]. What’s more, Cheng-Chieh Chang et al. found that tanshinone IIA (1 ~ 20 µM) inhibited the adhesion of THP-1 monocytes to HUVECs in response to TNF-α stimulation by downregulating IKK/NF-κB mediated VCAM-1, ICAM-1 and fractalkine expression in HUVECs [105]. There is a kind of polyphenol, quercetin, exerts anti-inflammatory effects and contributes to progression of atherosclerosis [106]. There is increasing evidence that both in hypercholesterolemic diet-induced rabbits and high-fat diet fed ApoE^−/−^ mice, quercetin is effective in slowing the progression of atherosclerosis [107, 108]. Similarly, another study demonstrated that in vitro and in vivo, quercetin reduced both VCAM-1 and E-selectin expression, as well as IKK gene expression implicated in local vascular inflammation, with a significant reduction (40%) in the atherosclerotic plaque [109].

Myricetin, also known as alias myricetin, myricetin, is a bark extract from *Myrica rubra Sieb. et Zucc*, has been found to have vascular protective properties [110]. With the development of medical research, the anti-inflammatory and anti-atherogenic properties of myricetin have been reported successively [111–113]. It has been shown that myceritin significantly reduced the plaque area in the aortic root of LDLR^−/−^ mice, as well as improved ox-LDL-induced cholesterol accumulation in macrophages in these mice [114]. Furthermore, according to an early study, myricetin inhibit monocyte adhesion to TNF-α-mediated ECV304 cells (a type of HUVECs) by strongly inhibiting IKK and its downstream signaling NF-κB/IκB [115]. The root of *clematis mandshurica* is used as anti-inflammatory agent in Chinese pharmacopoeia [116]. Clematichinenoside (a triterpene saponin), extracted from the root of clematis mandshurica, is beneficial in the early stage of atherosclerosis [117]. According to a study, clematichinenoside inhibits VCAM-1 and ICAM-1 expression in TNF-α-treated ECs via the NADPH oxidase-dependent IκB/NF-κB pathway [118].

There is an active bioactive diterpene lactone called andrographolide (AP) ectracted from *andrographis paniculata*, which has the biological functions, including anti-inflammation, anti-atherosclerosis, and hypoglycemic activities [119]. It is clear from a study that AP is a novel NF-κB Inhibitor, which inhibits the proliferation of VSMCs in atherosclerosis [120]. Another study showed that AP downregulated ICAM-1 expression in TNF-α-treated EA.hy926 cells (HUVECs fusion cell), at least partly by reducing the activation of IKK, indicating a cardioprotective role. Avenanthramide-c, a unique soluble polyphenol, is extracted from oats [121]. As a result of oat bran diets, atheroma lesions are reduced, and high levels of avenanthramides further reduce aortic lesions [122]. An immunofluorescence assay showed that avenanthramide-c reduced the translocation of NF-κB from the cytoplasmic region to the nucleus, and down regulated the expressions of IκB and p-IκB in TNF-α activated human arterial smooth-muscle cells (HASMCs) [123]. Moreover, avenanthramides, a unique polyphenol from oats, decreased the IL-1β-induced proinflammatory cytokines, such as IL-6, IL-8, and MCP-1, in human aortic endothelial cells (HAECs), at least in part by blocking IKK phosphorylation [124].

Cardiac glycoside digitoxins are natural steroid compounds originally exacted from *Digitalis sp,* there is strong evidence that cardiac glycoside digitoxin is a potent anti-inflammatory agent [125]. Digitoxin inhibits monocyte adhesion to endothelial monolayers, which is associated with inhibiting the IL-1β-induced NF-κB signaling at the level of TAK-1/IKK [126]. Kansuinine A is extracted from *Euphorbia kansui L.*, a well-known medicinal plant in China [127]. There is a study that confirms the anti-atherosclerotic properties of Kansuinine A by inhibiting the IKKβ/IκBα/NF-κB signaling in atherogenic animals and H_2_O_2_-stumilated HAECs [42, 128]. Honokiol is a small-molecule polyphenol that is extracted from the Chinese herbal medicine *Magnolia officinalis*, which has a number of pharmacological properties [129]. There is overwhelming evidence that honokiol suppresses inflammation and oxidative stress in the carotid arteries, inhibiting the formation of atherosclerotic plaque [130]. Surprisingly, in palmitic acid-inducted HUVECs, the expression of NF-KB subunits (p50 and p65), as well as IκB phosphorylation in the IKK/IκB/NF-κB signaling, was significantly inhibited by honokiol [90, 131].

Longxuetongluo Capsule (LTC) is a new drug consisting of the total phenolic extract of Chinese dragon blood. It is believed that Longxuetongluo capsules inhibit monocyte adhesion to the HUVECs through the MAPK/IKK/IκB/NF-κB signaling, thereby reducing atherosclerotic lesions in the aortic sinus of ApoE^−/−^ mice. Pulvones A and C were newly discovered isoflavones from *Millettia pulchra*, a renowned anti-inflammatory herbal medicine from southeast China [132]. In LPS-stimulated RAW264.7 macrophage cells, pulvones A and C decreased IL-6 and IL-1β expression, reduced the nuclear translocation of NF-κB (p65), and interrupted IκB phosphorylation by directly inhibiting the IKKβ kinase activity (40% inhibition), all of which were validated by docking studies [133].

Acetyl-11-keto-β-boswellic acid (AKBA), the main pharmacological component of Boswellia extract, is considered to be a natural inhibitor of the pro-inflammatory transcription factor NF-κB, exerting powerful anti-inflammatory and antioxidant effects [134]. As a result, AKBA significantly downregulated many NF-κB-dependent genes, including MCP-1, MCP-3, MIP-2, IL-1, VEGF and tissue factor (TF), as well as IKK activity, and resulted in a significant 50% reduction in the size of atherosclerotic lesions in LPS-injected apoE^−/−^ mice; furthermore, similar anti-inflammatory effects were found in LPS-stimulated mouse macrophages and mononuclear cells as well as human macrophages [135]. Ginsenoside Re, a major pharmacological active ingredient of ginseng, has been reported to be a potential therapeutic molecule for atherosclerosis and one of the most promising IKK-β inhibitors [136]. Ginsenoside Re inhibited IKKβ phosphorylation, NF-κB activation, and the expression of proinflammatory cytokines TNF-α and IL-1β in LPS-stimulated peritoneal macrophages, but had no effect on TNF-α-stimulated peritoneal macrophages [137].

Natural pentacyclic triterpenoids (PTs), ursolic acid (UA), and corosolic acid (CA) exhibit a wide range of biological activities, such as anti-inflammatory and cardio-protective effects, which are closely related to particularly the regulation of the NF-κB signaling pathway [138]. According to a hotspot kinase assay and in vitro experiments, UA and CA inhibited IKKβ and down-regulated the proteins expression of IKKβ/NF-κB cascade in LPS-stimulated RAW 264.7 cells, indicating that IKKβ is the main target of PTs-induced NF-κB inhibition [139]. Black pepper (*Piper nigrum L.*) is commonly used in cooking and traditional medicine in several countries and has been shown to be beneficial in atherosclerosis [140]. Pipernigramides (42–44), a new piperic ester isolated from black pepper EtOH extract, significantly inhibited inducible nitric oxide synthase (iNOS)-mediated release of NO, IL-1β, IL-6, TNF-α, and PGE2 in LPS-stimulated RAW 264.7 cells by targeting IKK-β [141].

### Synthetic drugs

Sulforaphane (SFN) is a phytocompound belonging to the isothiocyanate family isothiocyanate derived from cruciferous vegetables, such as broccoli [142]. The aortic histopathologic examination confirmed that SFN significantly reduced the expression of NF-κB in the aortic tissue of fed high cholesterol diet (HCD) rabbits [143]. Due to the inhibition of RhoA/ROCK/NF-κB signaling in human endothelial cells ECV-304, SFN attenuated TNF-α-induced ICAM-1 expression, as well as IKK phosphorylation, suggesting a beneficial role in the atherosclerosis-related inflammation [144–146]. Furthermore, SFN also downregulated endothelial lipase expression by inhibiting NF-κB in the same cellular model, which favored HDL cholesterol levels [91]. A hydrophilic vitamin obtained through diet, vitamin C, also known as ascorbic acid, is synthesized by all plants and most animals [147]. Excitingly, according to a clinical study, supplementing with vitamin C can prevent atherosclerosis by improving vascular reactivity and structure in passive smokers [148]. furthermore, vitamin C inhibits NF-κB activation by activating p38 mitogen-activated protein kinases in ECV304 and HUVECs induced by IL-1, PMA, H_2_O_2_, TNF, and IFN-γ [149].

As a micronutrient, zinc is essential for human health, which plays a variety of biological roles, such as aiding in growth, metabolism, and immunity [150]. There are evidence that zinc deficiency has a negative role in atherosclerosis in both animal studies and epidemic research [151]. *Prasad* et.al found that zinc increased A20 and A20–TNF-receptor associated factor-1 complex, decreased inflammatory cytokines by the IKKα/NF-κB signaling pathway, downregelated in HL-60, HUVECs, and SW480 cell lines [152]. 1-deoxynojirimycin, a unique polyhydroxy alkaloid, is the main active component of mulberry (*Morus indica L*.) leaves and has been found to prevent coronary heart disease (CHD) at least in part by inhibiting the IKK/NF-κB pathway [153]. Similarly, a placebo-controlled, double-blind clinical trial clarifies how 1-deoxynojirimycin does attenuate atherosclerotic lesions in patients with coronary heart disease [153]. Ebselen is a synthetic, organo-selenium radical scavenger compound that functions similarly to glutathione peroxidase [154], which exerts antiatherogenic effects by modulating the transcription factors NF-κB [42].

Polyethylene glycol-superoxide dismutase is an important modifier of SOD that protects ECs [155]. Prostaglandin A1, an anti-inflammatory cyclopentenone prostaglandin, is biosynthesized via dihomo-γ-linolenic acid. Treatment with polyethylene glycol-superoxide dismutase and prostaglandin A1 prevented homocysteine-induced activation of IKK kinase and NF-κB in HUVECs and HAECs [156]. Fatty acid binding protein (FABP) 4/5 is predominantly expressed in macrophages and/or adipocytes and plays essential roles in energy metabolism, inflammation and atherosclerosis [157]. A previous study in patients with angiographically proven coronary artery disease (CAD) showed that FABP 4 plays a critical role in the activation of mononuclear cells and the dysfunction of ECs in atherosclerosis. Interestingly, FABP 4/5 inhibitors, such as compounds A16 and B8, apparently reduced the levels of TNF-α and MCP-1 by inhibiting the  IKK/NF-κB pathway, exhibiting anti-inflammatory effects in LPS-stimulated RAW264.7 macrophages [157].

Early reports demonstrated that 8-tosylamino quinolone, a kind of a representative IKK inhibitor (BAY11-7082) analog, has anti-atherogenic effects [158]. Further studies revealed that BAY11-7082 diminished NO, TNF-α, IL-1β, IL-6, and PGE2 production, as well as NF-κB and IKK activation in LPS-activated RAW264.7 cells and peritoneal macrophages in a dose-dependent manner by inhibiting the Akt/IKK/NF-κB pathway [159]. In vivo, losartan, an angiotensin converting enzyme inhibitor, was found to significantly attenuate aortic atherosclerosis, inhibit ER stress, and reduce aortic inflammation in uremic apoE^−/−^ mice; in vitro, losartan inhibited the upregulation of GRP78 in Ang II-stimulated RAW264.7 macrophages and IKK and IκB phosphorylation [70]. It has been suggested that losartan has a protective effect against atherosclerosis in patients with uremic symptoms.

TMP195, a class IIa histone deacetylase inhibitor, reduced the characteristics of plaque vulnerability, thereby enhancing plaque stability in advanced lesions. In addition, transcriptional profiling studies revealed that TMP195 reduced expression of target genes of NF-κB in advanced lesions by inhibiting IKKβ [68]. 9-(2-chlorobenyl)-9H-carbazole-3-carbaldehyde (LCY-2-CHO), an agonist of NRF2, inhibited the inflammatory responses in cultured rat aortic VSMCs. By inhibiting IKK phosphorylation and IκBα degradation, LCY-2-CHO reduced IL-1β-induced inflammatory mediators, such as cyclooxygenase-2 (COX-2) and IL-8 [160]. Based on its anti-inflammatory properties in VSMCs, LCY-2-CHO has therapeutic potential in atherosclerosis [160].

### Other drugs

Human ß-defensin 3 (hBD3) is a cardio-protective natural peptide found in mucous membranes, cells of the epithelium, and cells of the endothelium. In ApoE^−/−^ mice, hBD3 inhibited atherosclerosis progression and suppressed *P.gingivalis* LPS-induced NF-κB activity [161]. What’s more, HBD3 reduces TNF-α-induced inflammation and monocyte adhesion in HUVECs with a dose-dependent effect by decreasing the phosphorylation of IKK-α/β, IκB and p65 subunit [162]. Similarly, glucagon-like peptide 1 (GLP-1) has been shown to be one of the incretin hormones, confers protection against atherosclerosis and myocardial injury [163–165].

The Mediterranean dietary is a plant-based, antioxidant-rich, unsaturated fat dietary pattern with lower cardiovascular diseases morbidity and mortality [166]. Whether a mediterranean diet with coenzyme Q (CoQ), 200 mg/day in capsules, contains 15% of energy as protein, 47% of energy as carbohydrate, and 38% of total energy as fat (24% MUFA provided by virgin olive oil, 10% saturated fatty acid, and 4% polyunsaturated fatty acid), affected the inflammatory response genes in elderly individuals was investigated. This dietary pattern reduced postprandial expression of p65 and IKKβ, suggesting anti-inflammatory activity [167].

Inflammatory responses can also be triggered by other stimuli such as TNFα, ox-LDL and Ang II on macrophages. Jianpi Huazhuo Tiaozhi granules (JHTG), a prepared Chinese herbal medicine, including dangshen, poria cocos, tangerine peel, towel gourd, amomum villosum, lotus leaf, atractylodes macrocephala, coix seed, wood fragrance, salvia miltiorrhiza, malt, hawthorn, and fried alisma orientalis, is commonly used clinical practice for the prevention of atherosclerosis [62]. Studies have shown that JHTG attenuates oxidative stress injury induced by ox-LDL in RAW264.7 macrophages, reducing the levels of ROS, the expression of NOX4, IKK-α, IKK-β, and NF-κB by blocking the NOX/ROS-NF-κB pathway [62].

Despite the widespread use of percutaneous coronary intervention (PCI) to treat coronary artery diseases, post-operative arterial restenosis remains a concern [168]. Fufang-Zhenzhu Tiaozhi Capsule (FTZ) is a chinese herbal medicine prescription including *rhizoma* coptidis, radix *salvia miltiorrhiza*, radix notoginseng, *fructus* ligustri lucidi, herba cirsii jeponici, cortex eucommiae, fructus citri sarcodactylis, and radix atractylodes macrocephala. Excitingly, FTZ reduces restenosis by inhibiting NF-κB activity and downregulating inflammatory factor expression in the atherosclerotic lesion of a rabbit restenosis model [76, 169]. It is well known that coronary atherosclerosis is the pathological basis for ischemic heart disease.

## Therapeutic potential and future considerations

The compelling evidence has demonstrated the contributory role of IKKβ/NF-κB signaling in the pathogenesis of atherosclerosis. Therefore, the IKKβ is very attractive and promising as a target for the treatment of atherosclerosis. This review expounds on the link between key cellular components of atherosclerosis and IKKβ. It supports the view that targeted inhibition of IKKβ may produce a beneficial effect in preventing atherosclerosis. As a result, inflammation-reducing drugs targeting IKKβ have been developed and applied in several cellular studies and animal models, including natural products-based derivatives, natural extracts, synthetic drugs, as well as peptides et.al other drugs (Table 1). As a matter of fact, we also need to take attention to the potential side effects of these drugs, for example, digestive side effects, such as abdominal pain, nausea, and vomiting, have been observed with vinpocetine [170], metformin [171], andrographolide [172], digitoxin [173], acetyl-11-keto-β-boswellic acid [174], ursolic acid [175], and liraglutide [176]. Additionally, it has been shown in repeated studies that green tea polyphenols [177], acetyl-11-keto-β-boswellic acid [174], and ursolic acid [175] cause liver damage/degeneration, while methyl-β-cyclodextrin [51] (parenteral administration), quercetin [109], and corosolic acid [139] have nephrotoxic potential. Andrographolide [172] and digitoxin [173] cause chest tightness, palpitations, and arrhythmic. Similarly, vinpocetine [170] and andrographolide [172] cause the symptoms including dizziness headache, convulsions, and coma. The side effects of the above mentioned drugs are in fact very difficult to avoid and therefore scientific use of medication is a must.

**Table 1:** The Role of various natural products and the derivatives in atherosclerosis through Inhibiting IKKβ-mediated Inflammation

Classification/Name	major ingredient	Drug Class	Drug Source	Effective Dose of Drug	Animal/Cell Model Induction	Effect	Target		Pathway	Ref
**Natural products-based derivatives**										
Vinpocetine	Vinpocetine	Vincamine alkaloid	Vinca minor	5 mg/kg (i.p.)	high cholesterol diet (16.6% fat, 10.6% sucrose and 1.3% cholesterol) for 12 weeks + apoE^−/−^ mice-induced atherosclerosis model	anti-inflammatory, anti-oxidant stress, inhibit monocyte adhesion to ECs	TNF-α, IL-6, MCP-1, MCP-1, MMP-2, MMP-9**↓**	p-Akt, p-IKKα, p-IKKβ, p-IκBα ↓	Akt/NF-κB	[80]
				30, 50 µM	20 μg/ml ox-LDL + PMA-induced macrophages for 12 h					
					20 μg/ml ox-LDL + (HUVECs + THP-1 cells) to induce monocyte-endothelial cell adhesion					
Metformin	guanidine	hypoglycemic agents	Galega officinalis	200 mg/kg/day (p.o.)	atherogenic diet (0.8% cholesterol and 3% soybean oil) for 10 weeks + new zealand white rabbits to induce atherosclerosis model	anti-inflammatory, inhibit monocyte adhesion to ECs	MCP-1, CRP, TNF-α, IL-6, IL-4, IL-10, IL-8, IL-1, VCAM-1, ICAM-1, LOX-1 ↓	p65 ↓, IκBα ↑	-	[86]
				100, 200, 300 µM/ml	10 ng/ml TNF-α + rabbit endothelial cells + rabbit monocytes					
				100, 500,1000 µM	10 ng/ml TNF-α + HUVECs-induced inflammation	anti-inflammatory	IL-6 ↓	P-IKKα/β↓, IκBα, P-AMPK, αAMPK ↑	PI3K/AMPK/IKKα/β	[88]
				10^−3^ mol/L	(10^−4^ mmol/L Insulin + 30 mM glucose + 1 µM dexamethasone) + HUVECs-induced Insulin resistance of endothelial cells model	enhance endothelial function	NO, eNOS, ET-1 ↑	-	-	[84]
Naringin	Naringin	flavonoid	grape, citrus species, fractus aurantii	50, 100, 200 μg/ml	10 ng/ml TNF-α stuimilated-HUVECs + THP-1- induced inflammation and monocyte adhesion	anti-inflammatory, inhibit monocyte adhesion to ECs	ICAM-1,VCAM-1, MCP-1, E-selectin, RANTES↓	p-p65, p-IκBα, p-IKKα/β ↓, IκBα ↑	IKK/NF-κB	[92]
Emodin	Emodin	anthraquonoid compound	dried root of Rhei Rizoma	10 µM	100 ng/ml Light + monocyte-like cell line (THP-1) to induce monocytes migration	anti-inflammatory, inhibit migration	CCR1, CCR2, ICAM-1, IL-8, MCP-1, TNF-α, IL-6 ↓	p38, p-p38, p-IκBα	p38/NF-κB	[94]
				10 µM	10 ng/ml TNF-α inducing rat aortic VSMCs migration	anti-inflammatory	MMP-2, MMP-9, MCP-1, IL-1β, IL-6, ICAM-1, VCAM-1↓	-	NF-κB pathway	[95]
Green tea polyphenols	Epigallocatechin gallate	polyphenols	green tea	0.1, 0.2, 0.4 mg/ml	50 mg/ml ox-LDL + HUVECs-induced inflammation model	anti-inflammatory, improve endothelial dysfunction	TNF-α↓	p65↓	IKK/NF-κB	[99]
Stybenpropol A	Stybenpropol A	phenylpropane derivative	Benzoinum	50, 200 μmol/ L	12.5 ng/ml TNF-α + HUVECs-induced inflammation	anti-inflammatory, anti-apoptotic	sVCAM-1, sICAM-1, IL-1β, IL-8, Caspase-9, Bax↓, Bcl-2 ↑	IKKβ, IκBα ↑	IKK/NF-κB	[100]
Methyl-β-cyclodextrin	Methyl-β-cyclodextrin	cyclic oligosaccharide	starch hydrolysis enzymatic	5 nM	(1 μg/ml LPS / 50 μg/ml ox-LDL) + (HUVECs + THP-1)-induced monocyte-endothelial adhesion	inhibit monocyte adhesion to ECs	ICAM-1, VCAM-1↓	p-p65, p65, IKK, Akt↓, IκB, p-Akt↑	LPS/IKK/NF-κB, oxLDL/Akt/NF-κB	[51]
**Natural extracts**										
Tanshinone IIA	Tanshinone IIA	Diterpene	Salvia miltiorrhiza bunge	90, 30 10 mg/kg/day (p.o.)	high-fat diet (fat: 21% (wt/wt), cholesterol: 0.15% (wt/wt)) for 13 weeks + ApoE^−/−^ mice-induced atherosclerosis model	anti-inflammatory	MC-1, TNF-α ↓	TLR4, MyD88, NF-κB ↓	TLR4/MyD88/NF-κB	[104]
				5, 10, 20 µg/ml	10 ng/ml TNF-α + (HUVECs + THP-1)-induced inflammation	anti-inflammatory	ICAM-1,VCAM-1, TNF-α, E-selectin ↓	IKKα/β, p-IKKα/β, p65, p-IκBα, p-p65↓, IκBα↑	IKK/NF-κB	[105]
Quercetin	Quercetin	Flavonoid subclass	fruits and vegetables	0.1%, w/w in diet	human CRP transgenic mice + IL-1β 100 L/mouse (i.p.); ApoE*3 leiden transgenic mice + high cholesterol diet (15% (w/w) cacao butter, 1% (w/w) palm oil, 40.5% sucrose, 20% acid casein, 10% corn starch and 6.2% cellulose, supplemented with or without 1% cholesterol (all w/w) for 15 weeeks-induced AS model	anti-inflammatory, anti-proliferation	CRP, SAA, E-selectin, VCAM-1 ↓	p65	-	[109]
				10, 30 µmol/L	0.2 mmol/L H_2_O_2_/10 U/ml TNF-α/IL-1β 5 ng/ml- stimulated HUVECs/human HuH7 hepatoma cells					
Myricetin	myricetin	flavonoid	fruits, vegetables, medicinal herbs	-	TNF-α + ECV304 cells-induced inflammation	inhibit monocyte adhesion to ECs	-	IKK, p65, IκB ↓	IKK/NF-κB/IκB	[115]
				10 µgl/ml	10 ng/ ml LPS + dendritic cells-induced cells inflammation model	anti-inflammatory	TNF-α, IL-6, IL-12p70 ↓	P65, p-IKKα/β, p-IκBα ↓, IκBα ↑	IKK/NF-κB	[178]
Clematichinenoside	Clematichinenoside	triterpene saponin	Clematis chinensis osbeck root	1, 3, 10 µM	10 ng/ml TNF-α + HUVECs-induced inflammation	anti-oxidant stress, suppress monocyte- HUVECs adhesion	ICAM-1,VCAM-1, ROS, O2(∙-), H_2_O_2_, NOX4, p47↓	p-IκBα, IKKβ, p65↓, IκBα ↑	IKK/NF-κB	[118]
Andrographolide	Andrographolide	diterpene lactone	leaves	10 μM	1 ng/ml TNF-α + EA.hy926 cells -induced inflammation	anti-inflammatory	ICAM-1 ↓	p-IKKβ/IKKβ, p-IκBα, p-IKKα/IKKα, p65↓, IκBα ↑	IKK/NF-κB	[179]
Avenanthramide-c	CH_3_-Avenanthramide-c	polyphenol	Avena sativa	50, 100 μM	100 ng/ml TNF-α + human aortic SMCs to induce proliferation and migration	anti-inflammatory, anti-proliferation	MMP-2, MMP-9, TNF-α, IL-1β, IL-6 ↓	p65, p-IκB, p-ERK1/2, p-JNK, p-p38↓, IκB ↑	MAPK/NF- κB	[123]
				20, 40, 100 μM	5 ng/ml hrIL-1β + HAECs / 20 ng/ml TNF-α + HUVECs-induced inflammation	anti-inflammatory	IL-6, IL-8, MCP-1↓	p65, p-IKKα, p-IKKβ, p-IκB↓, IκBα↑	IKK/NF-κB	[124]
Digitoxin	digitoxin	steroid glycoside	Digitalis	3, 10, 30 nmol/l	10 ng/ml IL-1β + HUVECs-induced inflammation	anti-inflammatory	MCP-1, Caspase-3, eNOS↓	p65, p-TAK1, p-IKK, p-IκBα ↓, IκBα↑	TAK-1/IKK/NF-κB	[126]
Kansuinine A	Kansuinine A	Terpenoid	Euphorbia kansui L	20, 60 μg/ kg (i.p.)	A high fat-diet + ApoE^−/−^ mice + -induced atherosclerosis model	anti-oxidant stress, anti-apoptotic, anti-inflammatory	ROS, GPx, MDA, Bax, Bcl-2, CC3 ↓	p-p65, p65, p-IKKβ, IKKβ, p-IκBα↓, IκBα ↑	IKKβ/IκBα/NF-κB	[128]
				0.1, 0.3, 1.0 μM	200 μM H_2_O_2_ + HAECs-induced endothelial injury					
Honokiol	Honokiol	pleiotropic lignan	Magnolia grandiflora	10 μM	0.5 mM Palmitic acid (PA) + HUVECs-induced endothelial cell injury model	anti-inflammatory	IL-6, IL-8, MCP-1, NO, iNOS, eNOS ↓	p-IKKβ, p-IκB, p50, p65↓	IKK/IκB/NF-κB	[131]
Longxuetongluo capsule	Dragon's Blood	totaenolic extract	Croton, Dracaen, Daemonorops, Pterocarpus	100, 200, and 300 mg/kg/d (p.o.)	A high-fat diet (0.2% cholesterol,15% fat added) for 7 weeks + ApoE^−/−^ mice-induced atherosclerosis model	anti-inflammatory, inhibit the adhesion of monocytes to HUVECs	eNOS ↑, VCAM-1, MCP-1, COX-2 ↓	p-ErK/ErK, p-IKKα/β, p-IκBα, IKKβ, IKKα, p-p38/p38↓, IκBα ↑	p38/IKK/IκB/NF-κB	[180]
				20, 40 µg/ml	20 µg/ml ox-LDL + (HUVECs + THP-1)-induced inflammation					
Pulvones A/C	pulvones A/C	isoflavonoids	*Millettia pulchra*	3, 10 µM	1 µg/ml LPS + RAW264.7 macrophage cells to induce inflammation	anti-inflammatory	iNOS, COX-2, IL-6, IL-1β↓	p65, IKK, p-IκBα↓, IκBα↑	IKKβ/NF-κB	[133]
Acetyl-11-Keto-β-Boswellic Acid	Acetyl-11-Keto-β-Boswellic Acid	γ-cyclodextrin complex	oleogum resin	100 mol/kg (i.p.)	50 μg LPS-injected apoE^−/−^ mices-induced atherosclerotic model	Anti-inflammatory anti-atherogenic	MCP-1, MCP-3, IL-1α, MIP-2, VEGF, TF ↓	IKK, p-IκBα, p65↓, IκBα↑	NF-κB signaling	[135]
				10 μmol/L	100 ng/ml LPS + human macrophages / mouse mononuclear and macrophages-induced inflammation model					
Ginsenoside Re	Ginsenoside Re	-	Ginseng	10 µM	50 ng/ml LPS + peritoneal macrophages to induce inflammation model	anti-inflammatory	IL-1β, TNF-α, COX-2, iNOS, IRAK-4, IRAK-1↓	p-IKKβ, p65, p-p65, TLR4, MyD88 ↓, IκBα ↑	-	[137]
Corosolic acid	Corosolic acid	Pentacyclic triterpenoid	-	50 μM	1 μg/ml LPS + RAW 264.7 macrophage cells -induced inflammation model	anti-inflammatory	IFN-γ ↓	Akt, NF-κB, c-JUN, IKKα, p-IKKα, p-IKKβ ↓	Akt/IKK/NF-κB	[139]
Ursolic acid	Ursolic acid			100 μM						
Pipernigramides	compounds 42/43/44	amide alkaloids	*Piper nigrum L*	2, 4, 8 μM	1 μg/ml LPS + RAW 264.7 macrophage cells -induced inflammation model	anti-inflammatory	IL-1β, IL-6, TNF-α, PGE_2_, iNOS, COX-1, COX-2 ↓	IKKα/β, P-IKKα/β, p-IκBα, p65, p-p65 ↓, IκBα ↑	NF-κB signaling	[141]
**Synthetic drugs**										
Sulforaphane	Sulforaphane	isothiocyanate	Isothiocyanate, broccoli	0.25 mg/kg/day (p.o.)	A high cholesterol die (1% cholesterol-enriched chow) for 4 weeks + new zealand white rabbits to induce atherosclerosis model	anti-oxidant stress	MDA, SOD ↓, GSH ↑	p65	-	[143]
				2.5, 5 μM	5 ng/ml TNF-α + Human endothelial cells ECV-304-induced inflammation	anti-inflammatory	IL-1β, IL-6, IL-8, ICAM-1, VCAM-1, E-select ↓	p65, p-IκBα, p-IKKβ, IKKβ, p-RhoA, RhoA, p-ROCK, ROCK↓, IκBα↓	RhoA/ROCK/NF-κB	[146]
				10 μmol/L	4 ng/mL TNF-α + HUVECs to induce inflammation model	anti-inflammatory	EL ↓	p50, p65, p-IKK1/IKK1, p-IKK2/IKK2, p-IκB/IκB↓, IκBα ↑	NF-κB/EL	[91]
Vitamin C	Vitamin C	Vitamin		10, 20, 40 mM	10 ng/ml IL-1/100 ng/ml PMA / 0.2 mM H_2_O_2 /_ 10 ng/ml TNF / 200 U/ml IFN-γ + ECV304 / HUVECs-induced inflammation model	anti-inflammatory anti-oxidant stress	IL-8 ↓	p65, p-IκBα, IKKα, IKKβ ↓, IκBα ↑	-	[149]
Zinc	Zinc	-	-	1, 15 μM	10 μg/ml LPS + HUVECs-induced inflammation model	anti-inflammatory	TNF-α, IL-1β, MCP-1, NO, VCAM-1 ↓	IKKα, p65↓	IKKα/NF-κB	[152]
1-Deoxynojirimycin	1-Deoxynojirimycin	Polyhydroxy alkaloid	Mulberry Leaves	10 mg/d (p.o.)	A total of 144 patients with stable angina pectoris and blood stasis syndrome	anti-oxidant anti-inflammatory	hs-CRP, IL-6, TNF-α, SOD, MDA ↓	IKK, p65↓, IκBα ↑	-	[153]
Ebselen	2-phenyl-1,2-benzisoselenazol-3[2H]-one]	organoselen-ium compound	Synthetic compound	20 mg/kg/d	C57Bl/J6 apoE^−/−^ mice + streptozotocin-induced diabetic apoE^−/−^ mouse model	anti-atherosclerosis	VEGF, RAGE, N_OX_2, SOD-1, GPx1, catalase, TNF-α ↓	p-IKK, p-JNK↓	-	[42]
				0.03 μmol/L	100 μmol/L H_2_O_2_ + HAECs-induced inflammation model					
Polyethylene glycol-superoxide dismutase	-	superoxide anion scavenger	-	300 U/mL	100 μmol/L homocysteine + HUVECs / HAECs to induce endothelial injury	anti-oxidant stress, anti-inflammatory	SOD ↓	p-IκBα, IKKα, IKKβ, p65↓, IκBα ↑	-	[156]
Prostaglandin A1	-		-	30 μmol/L						
FABP 4/5 inhibitors	Compound A16/B8	FABP 4/5 inhibitor	-	30, 60 µM	100 ng/ml LPS + RAW 264.7 macrophage cells -induced inflammation model	anti-inflammatory	MCP-1, TNF-α, IL-6, COX-2↓	IKK, p-IKK, p65, p-p65 ↓	IKK/NF-κB	[181]
BAY11-7082 analogs	8-tosylamino quinoline	IKK inhibitor	-	20 μmol/L	1 μg/ml LPS + RAW264.7 macrophage cells and peritoneal macrophages-induced inflammation model	anti-inflammatory	NO, PGE_2_, TNF-α, IL-12p40, iNOS, COX-2 ↓	p65, IKK, p-IKK, Akt, p-Akt, p-IκBα ↓, IκBα ↑	Akt/NF-κB	[159]
Losartan	Losartan	Ang II type 1 receptor antagonist	-	30 mg/kg	male apoE^−/−^ mice + 5/6 nephrectomy to induce experimental mild uremia	antiinflammatory anti-atherosclerosis	BUN, CRE, CCL2/MCP-1, CX3CL1, TNF-α, IL-6↓	IRE1α, p-IRE1α, GRP78, p-IKK, IKKα, IKKβ, p65 ↓, IκBα ↑	IRE1α/IKK/NF-κB	[70]
				10 μmol/L	1 μg/ml Ang II + RAW264.7 murine macrophages to induce inflammation model					
TMP195	TMP195	class IIa histone deacetylase inhibitor		50 mg/kg/d, i.p	ApoE^–/–^ mice + Western-type diet (21% fat) to induce atherosclerosis model	alleviate vascular inflammation	CXCL-1, CCL-2, TNF-α, IL-1β, IL-6, IL-8, VCAM-1 ↓	IKKβ, p-p65 ↓	IKKβ/NF-κB	[68]
				3, 5 µmol/L	50 ng/mL mouse recombinant + Human Peripheral Blood Mononuclear Cells (PBMCs) to induce inflammation model					
LCY-2-CHO	LCY-2-CHO	carbazole analogue	-	3, 10 μM	50 nM TNF-α/10 ng/ml IL-1β + Rat aortic VSMCs to induce inflammation model	anti-inflammatory	HO-1, Nrf2, Lamin B, iNOS, COX-2, IL-8, GRO-α, eNOS, p-eNOS, MBP ↓	IKK, p-IKK ↓, IκBα ↑	IKK/NF-κB	[160]
**other drugs**										
β-defensins 3	β-defensins 3	Antimicrobial peptide	mucosa and epithelial cells	15, 10, 5 μg/ml	40 ng/ml TNF-α + (HUVECs + TPH-1)-induced inflammation and monocyte adhesion	anti-inflammatory, reduce monocyte adhesion	IL-6, IL-8, MCP-1, MIF, ICAM-1, VCAM-1, Bax, E-selectin, ROS, cleaved caspase-3/caspase-3↓, Bcl-2 ↑	p-IKK, p-IκBα, p-p65↓, IκBα ↑	NF-κB, MAPK	[162]
Glucagon-like peptide 1	Liraglutide	incretin hormone	gut enteroendocrine cells	30 nM	5 ng/ml TNF-α + HUVECs-induced oxidative damage and inflammation	anti-oxidant stress, anti-inflammatory	SOD, catalase, GPx ↑	PKC-α, P-IKKα/β, IKKα, IKKβ ↓, IκBα ↑	-	[165]
				s.c and tail i.v 0.2 mg/kg	2.5 mg/kg adriamycin (i.p.) + GK rats for 6 weeks- induced diabetic cardiomyopathy model	anti-oxidant stress, alleviate myocardial fibrosis	MDA↓, SOD, GPx ↑	PPARγ↑, p65 ↓	-	[164]
Mediterranean diet + CoQ	-	-	-	Med diet + CoQ	Clinical inclusion criteria: age 65 years or older, body mass index 20 ~ 40 kg/m^2^, total cholesterol concentration ≤ 8.0 mmol/L, and nonsmokers	anti-inflammatory	IL-1β, JNK-1, MMP-9, sXBP-1, CRT, BiP/Grp78 ↓	p65, IKKβ ↓, IκBα ↑	-	[182]
Jianpi huazhuo tiaozhi granules	-	chinese herbal medicine	-	2.5%, 5%, 10% concentrations	100 mg/L ox-LDL + RAW264.7 macrophage cells- induced oxidative stress injury model	anti-oxidant stress anti-apoptosis	MDA, SOD, ROS, NOX4, p22phox **↓**	IKKα, IKKβ, p65↓	NOX/ROS/NF-κB	[62]
Fufang-Zhenzhu-Tiaozhi Capsule	-	Chinese herbal medicine	-	0.66 mg/kg/d	male new zealand rabbits + balloon rubbing the endothelium of the abdominal aorta + a high fat diet (1.5% cholesterol, 0.5% sodium cholate, 8% lard, and 10% egg yolk powder)-induced atherosclerosis model	lipid-lowering anti-inflammatory	TC, TG, LDL-C, VLDL-C, IL-1, IL-6, IL-8, IL-12, TNF-α, MCP-1, ICAM-1 ↓	p65, p-IκBα, IKK-α, p-IKKα/β↓, IκBα ↑	IKK/NF-κB	[169]

*i.p.* Intraperitoneally, *p.o.* Persral oral

In summary, as more drugs targeting IKKβ are discovered, there will be more opportunities to fully understand the complex functions of IKKβ in atherogenesis and to develop new effective therapies. Further result should be conducted in the future to enhance the understanding of drugs with potential therapeutic effects to treat atherosclerosis via IKKβ, such as additional validation experiments, comparative efficacy experiments among different drugs, and multicellular targeting experiments and clinical trials, etc. Understanding the pathogenesis of diseases associated with impaired IKKβ activity may provide insight into prevention and treatment of these human diseases.

## Data Availability

The declaration is not applicable.
